# Effect of Gap Distance on Tensile Strength of Preceramic Base Metal Solder Joints

**DOI:** 10.5681/joddd.2011.018

**Published:** 2011-09-05

**Authors:** Farnaz Fattahi, Milad Motamedi

**Affiliations:** ^1^Assistant Professor, Department of Prosthodontics, Faculty of Dentistry, Shiraz University of Medical Sciences, Shiraz, Iran; ^2^Postgraduate Student, Department of Prosthodontics, Faculty of Dentistry, Shiraz University of Medical Sciences, Shiraz, Iran

**Keywords:** Base metal, gap distance, porosity, solder, tensile strength

## Abstract

**Background and aims:**

In order to fabricate prostheses with high accuracy and durability, soldering techniques have been introduced to clinical dentistry. However, these prostheses always fail at their solder joints. The purpose of this study was to evaluate the effect of gap distance on the tensile strength of base metal solder joints.

**Materials and methods:**

Based on ADA/ISO 9693 specifications for tensile test, 40 specimens were fabricated from a Ni-Cr alloy and cut at the midpoint of 3-mm diameter bar and placed at desired positions by a specially designed device. The specimens were divided into four groups of 10 samples according to the desired solder gap distance: Group1: 0.1mm; Group2: 0.25mm; Group3: 0.5mm; and Group4: 0.75mm. After soldering, specimens were tested for tensile strength by a universal testing machine at a cross-head speed of 0.5mm/min with a preload of 10N.

**Results:**

The mean tensile strength values of the groups were 162, 307.8, 206.1 and 336.7 MPa, respectively. The group with 0.75-mm gap had the highest and the group with 0.1-mm gap had the lowest tensile strength. Bonferroni test showed that Group1 and Group4 had statistically different values (P=0.023), but the differences between other groups were not sig-nificant at a significance level of 0.05.

**Conclusion:**

There was no direct relationship between increasing soldering gap distance and tensile strength of the solder joints.

## Introduction

In order to fabricate prostheses with high accuracy and durability, soldering techniques have been introduced to clinical dentistry. Procedures such as long-span fixed partial dentures, repair of clasps and frameworks, addition of wrought wire clasps in removable prostheses, and the superstructures of dental implants all need soldering to improve accuracy and solve fitting problems.^1^ Two main methods are available for soldering:^2^preceramic and postceramic soldering, which are done before and after porcelain application, respectively. Preceramic soldering is often performed at a temperature very close to the melting point of parent metals, but postsolder melts below the pyroplastic range of the porcelain, which is considerably lower than the melting point of the parent metal.

However, these prostheses always fail at their solder joints. When assembling a prosthesis, it is frequently necessary to solder or weld some parts together, which is highly predictable when using noble metals.^3^ Soldering is the most common and practical procedure used to join fixed dental prostheses by using a metal (solder) with a melting point which is lower than the parent alloy.^4^Gegauff et al^5^ reported that soldering results in the best margin adaptation when compared to one-piece castings. Base metal alloys have some disadvantages, including high Brinell hardness, elevated casting temperatures, low specific gravities, and inconsistent soldering.^6^ Soldering of the base metal alloys is difficult because they have a potential for high oxidation at increased temperatures. Preceramic soldering of base metals may improve under controlled conditions such as an oven environment under vacuum.^7^ Controversy exists in the literature concerning the solder connector strength of fixed partial denture components. Problems related to soldering procedures vary according to the different methods and materials used. There are significant factors associated with successful soldering, which include alloy composition, solder material, surface contamination, gap distance, indexing method and material, wetting angle, the length and shape of solder connectors, surfaces to be soldered, soldering temperature, and the thickness of the retainers.^8 - 12^ Gap distance is one of the variables reported by several authors to influence the accuracy of soldering. It has been reported that the ideal gap distance ranges from 0.1 mm to 0.75 mm.^13 - 15^ Rasmussen et al^16^reported that increasing the gap width will increase the strength of the solder connectors. Another study found that a solder joint might be stronger when narrower gaps are used.^14^ One study showed that the gap distance does not have an effect on the solder connector strength.^8^ According to Stade et al^17^ the method of soldering is more significant in connector strength than the gap width. The purpose of this study was to evaluate the effect of gap distance on the tensile strength of base metal solder joints. Specimens were tested with the gap distances of 0.1 mm, 0.25 mm, 0.5 mm and 0.75mm.

## Materials and Methods

**Figure 1:**
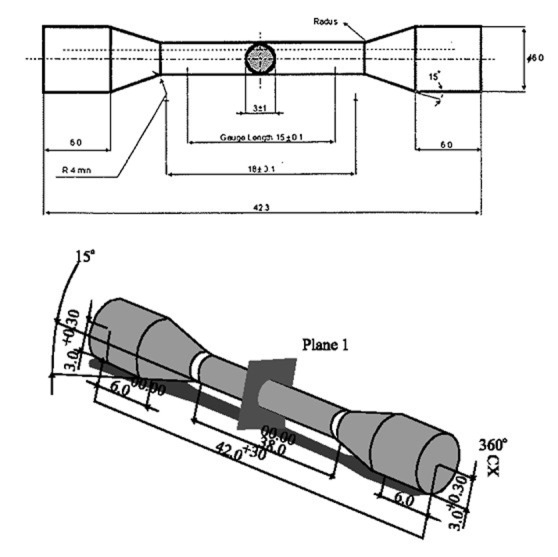
ADA/ISO 9693 model for tensile strength test

Based on ADA/ISO 9693 specifications for tensile test, a brass model was prepared (Figure 1),which included a 3-mm diameter bar which was to be cut and soldered in the middle.

**Figure 2:**
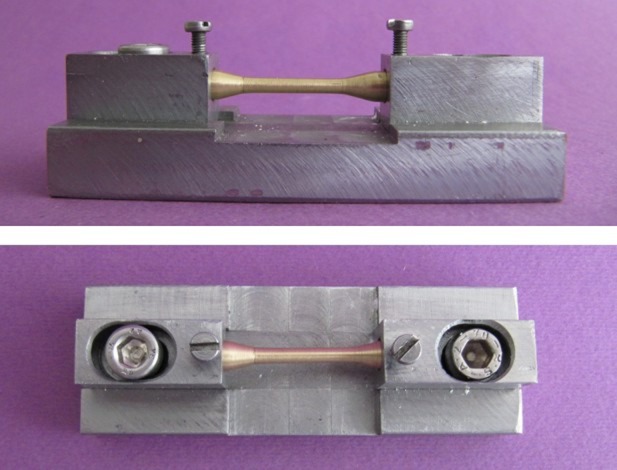
Specific device to hold the specimens at desired positions.

A silicon impression was made (Speedex, Coltene, Germany) from the model, and 40 wax models were prepared to fabricate 40 specimens. Spruing and investing (Ernst Hinrichs GmbH, Germany) were carried out and casting (Ni-Cr Alloy, 4All, Ivoclar Vivadent, Liechtenstein) was performed by a vacuum/pressure casting machine (Natilus T, Bego, Germany) according to manufacturer’s instructions. Sprues were cut by carborundum disc, and the specimens were finished and polished. A custom device was designed and fabricated to hold the specimens firmly while being cut and positioned at desired gap distances (Figure 2).

The specimens were cut by a 0.3-mm carborundum disc at the midpoint by a nonstop cutting machine; then an index gauge of desired gap distance (0.1 mm, 0.25 mm, 0.5 mm and 0.75 mm) was placed to determine the position to be soldered. The specimens were divided into four groups of 10 according to the solder gap distance: Group1: 0.1 mm; Group 2: 0.25 mm; Group 3: 0.5 mm; and Group 4: 0.75 mm. Soldering procedure was performed according to Rosenstiel’s recommended method.^2^ Pattern resin (GC America Inc.) was used to fix the specimens in desired positions while being invested and soldered. Resin was burned out by a torch at 300°C and any probable contamination was removed. Flux (High-fusing Bondal™ Flux, Ivoclar Vivadent, Liechtenstein) was applied and the specimens were put in the furnace. Temperature was gradually increased up to 850°C until the specimens became red. At that moment, the specimens were retrieved from the furnace, and placed on the soldering stand. A pinpoint flame of gas-oxygen torch was used at its reducing zone concentrated on the joint space and the solder was placed above the gap. After bench cooling for about 5 minutes, the assembly was ready to break the investment and evaluate the solders. The specimens were tested by hand force and then, a radiograph was taken to make sure that the solders are complete.^2 , 18^ Joint irregularities were ground away and finishing and polishing procedures was performed. In this study, the specimens were tested for tensile strength by a universal testing machine (Instron, Zwick20, Germany) at a cross-head speed of 0.5mm/min with a preload of 10N. The specimens were investigated after the test for defects, voids and location of the failure. Cross-sections were observed by a stereomicroscope (Motic, China) with the magnification scale of 40X. Data were collected and analyzed by SPSS software using one-way ANOVA and Bonferroni tests.

## Results

As shown in Table 1, the mean tensile strength values of the groups were 162, 307.8, 206.1 and 336.7 MPa, respectively. The group with 0.75-mm gap had the highest and the group with 0.1-mm gap had the lowest tensile strength.

**Table 1:** Tensile strengths of the samples

Group	N	Minimum(MPa)	Maximum (MPa)	Mean(MPa)	Std. Deviation
1	10	45.10	466.60	162.03	129.21567
2	10	37.70	459.60	307.84	148.16209
3	10	64.20	345.00	206.17	106.07186
4	10	131.10	522.00	336.75	117.24329

Data were analyzed by one-way ANOVA test, which revealed significant differences between the groups under study (P=0.011).

Multiple comparisons were carried out by Bonferroni test, which showed that Groups1 and 4 had statistically different values (P=0.023), which means solder joints with the gap distance of 0.75mm had higher tensile strength than those with 0.1-mm gap distance. However, the differences between other groups were not significant (P>0.05).

## Discussion

**Figure 3:**
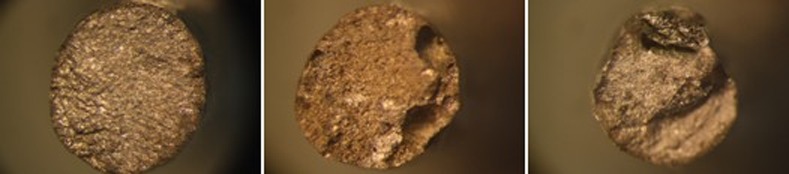
Relatively uniform solder versus solders with great voids (×40).

A disadvantage of preceramic soldering is the risk of parent alloy melting and also microporosity or pitting as a result of volatilization of base metal alloy constituents.^19^ Porosity in solder joints may be produced by flux entrapment due to over-fluxing and under-heating, in addition to wetting ability of molten solder in solder gap space (Figure 3). ^1 ,3 ,18^

**Figure 4:**
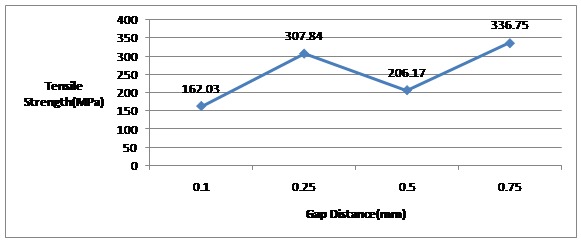
Relationship between gap distance and tensile strength.

Fixed partial prostheses commonly fracture clinically under high tensile forces; therefore, tensile strength test seems to be an appropriate method to evaluate the strength of solders.^20^ In this study, preceramic base metal soldering was performed according to manufacturers’ recommendations, and no consistent relationship was found between the gap distance increase and tensile strength of joints. Figure 4 shows that there is no direct relationship between the soldering gap distance and tensile strength.

This inconsistency might be attributed to solder wetting ability in gap space, solder material solidification shrinkage in different gap distances, and flux entrapment in solder space which can cause porosities resulting in decreased solder joint strength.^18^ All the specimens were broken in the solder area suggesting this area as the weakest point of the soldered assembly. In addition, high standard deviations in this study might be attributed to technique sensitivity of the procedure and probable human or material errors.

Rasmussen et al^16^associated the strength of solder joints with five general factors: wetting, strength of metals to be joined, geometry of the joint, flux inclusions and voids and thickness of soldering alloy in the gap. They concluded that different alloys will show different results as the soldering gap increases, which is consistent with the results of this study. They also reported that postsolder joints are significantly stronger than presolder joints. The high-fusing solders have lower surface tension which enables them to flow more easily into narrow gaps. In a study by Stade et al,^17^ a consistent rise was found in strength values as the solder gap distance increased. However, in the present study, this rise was not directly related to the gap distance. Results of this study support the findings of Lee et al,^20^ who did not report a relation between gap distances of 0.3mm and 0.5mm and the tensile strength of the solder joint. Willis et al^3^suggested using a minimum gap distance without contact in contrast to the present study, in which the group with 0.1-mm gap, showed the least tensile strength.

## Conclusion

This study evaluated the effect of gap distance on the tensile strength of preceramic base metal Nickel-Chromium solder joints. It was concluded that:

1. There was no direct relationship between increasing soldering gap distance and tensile strength of the solder joints.
2. All the specimens fractured at the solder joint space suggesting it as the weakest point of soldered assembly.
3. Porosities and voids found in specimens could be attributed to flux entrapment or solder inability to flow into the gap space.
